# Lesion-Specific Peri-Coronary Fat Attenuation Index Is Associated With Functional Myocardial Ischemia Defined by Abnormal Fractional Flow Reserve

**DOI:** 10.3389/fcvm.2021.755295

**Published:** 2021-11-03

**Authors:** Shaowei Ma, Xujiao Chen, Yue Ma, Hui Liu, Jiayin Zhang, Lei Xu, Yining Wang, Ting Liu, Kunhua Wang, Jinzhu Yang, Yang Hou

**Affiliations:** ^1^Department of Radiology, Shengjing Hospital of China Medical University, Shenyang, China; ^2^Department of Cardiology, Shengjing Hospital of China Medical University, Shenyang, China; ^3^Department of Radiology, Guangdong General Hospital, Guangzhou, China; ^4^Institute of Diagnostic and Interventional Radiology and Department of Cardiology, Shanghai Jiao Tong University Affiliated Sixth People's Hospital, Shanghai, China; ^5^Department of Radiology, Beijing Anzhen Hospital, Capital Medical University, Beijing, China; ^6^Department of Radiology, Peking Union Medical College Hospital, Chinese Academy of Medical Sciences and Peking Union Medical College, Beijing, China; ^7^Department of Radiology, The First Affiliated Hospital of China Medical University, Shenyang, China; ^8^Department of Radiology, The People's Hospital of Liaoning Province, Shenyang, China; ^9^Key Laboratory of Intelligent Computing in Medical Image, Ministry of Education, Northeastern University, Shenyang, China

**Keywords:** computed tomography angiography, coronary artery disease, adipose tissue, fractional flow reserve (FFR), functional ischemia

## Abstract

**Background:** The association between abnormal invasive fractional flow reserve (FFR) and the fat attenuation index (FAI) of lesion-specific peri-coronary adipose tissue (PCAT) is unclear.

**Method:** Data of patients who underwent coronary computed tomography angiography (CTA) and subsequent invasive coronary angiography (ICA) and FFR measurement within 1 week were retrospectively included. Lesion-specific FAI (FAI_lesion_), lesion-free FAI (FAI_normal_), epicardial adipose tissue (EAT) volume and attenuation was collected, along with stenosis severity and plaque characteristics. Lesions with FFR <0.8 were considered functionally significant. The association between FFR and each parameter was analyzed by logistic regression or receiver operating characteristic curve.

**Result:** A total of 227 patients from seven centers were included. EAT volume or attenuation, traditional risk factors, and FAI_normal_ (with vs. without ischemia: −82 ± 11 HU vs. −81 ± 11 HU, *p* = 0.65) were not significantly different in patients with or without abnormal FFR. In contrast, lesions causing functional ischemia presented more severe stenosis, greater plaque volume, and higher FAI_lesion_ (with vs. without ischemia: −71 ± 8 HU vs. −76 ± 9 HU, *p* < 0.01). Additionally, the CTA-assessed stenosis severity (OR 1.06, 95%CI 1.04–1.08, *p* < 0.01) and FAI_lesion_ (OR 1.08, 95%CI 1.04–1.12, *p* < 0.01) were determined to be independent factors that could predict ischemia. The combination model of these two CTA parameters exhibited a diagnostic value similar to the invasive coronary angiography (ICA)-assessed stenosis severity (AUC: 0.820 vs. 0.839, *p* = 0.39).

**Conclusion:** It was FAI_lesion_, not general EAT parameters, that was independently associated with abnormal FFR and the diagnostic performance of CTA-assessed stenosis severity for functional ischemia was significantly improved in combination with FAI_lesion_.

## Introduction

The presence of coronary artery disease (CAD), specifically the presence of a flow-limiting lesion, is a primary reason for the onset of myocardial ischemia. Invasive fractional flow reserve (FFR) is highly recommended to determine the drop in flow around a coronary lesion to guide decision-making for coronary revascularization (1, 2), but it is not considered appropriate for clinical screening. As a non-invasive imaging modality, coronary computed tomography angiography (coronary CTA) is widely used for the detection of obstructive CAD. CTA-derived fractional flow reserve (FFR_CT_) had been proved to significantly improve diagnostic accuracy for the detection of ischemia-causing lesions and is recommended to evaluate the functional significance of intermediate stenosis (30–90%) to help guide invasive coronary angiography (ICA) referral and revascularization treatment planning (3–5). However, according to the Computed Tomographic Evaluation of Atherosclerotic Determinants of Myocardial Ischemia (CREDENCE) trail, the addition of FFR_CT_ to plaque features (percentage of non-calcified atheroma volume, lumen volume, and high-risk plaque features) and stenosis severity did not improve the predictive ability of CTA on FFR abnormal (6). Meanwhile, lesion features measured by CTA such as the percentage of luminal stenosis, total plaque volume (TPV), low-attenuation plaque volume, and positive remodeling were demonstrated to be cofactors associated with abnormal invasive FFR (7–9), but there was still divergence about specific features among those studies. Epicardial adipose tissue (EAT) is a cardiac-specific visceral fat depot that showed a close connection to cardiovascular diseases (10–12), and its volume could be quantified by coronary CTA. However, prior works did not demonstrate its predictive value for FFR-assessed myocardial ischemia (13–16).

In the past few years, peri-coronary adipose tissue (PCAT) had been shown to be an updated image biomarker of CAD (17–21). Fat attenuation index (FAI) of PCAT has been shown to reflect vascular inflammation associated with unstable plaque features (19, 22), and the vessel-specific FAI measurement was considered as an inflammatory biomarker to improve cardiovascular risk discrimination (20). The vessel-specific FAI was shown to be connected with abnormal FFR values in the presence of any lesions (9, 23), whereas the non-lesion specific method might compromise its independent predictive value among other lesion features. As a parameter that reflected the focal inflammatory burden around specific lesions, the association between lesion-specific PCAT attenuation and abnormal FFR was investigated in limited studies. Yu et al. indicated that lesion-specific PCAT attenuation could help to identify ischemic lesions (24), while the results of Du et al. showed that there was no connection between lesion-specific PCAT attenuation and abnormal FFR (16).

This study was conducted to investigate the association between lesion-specific FAI and abnormal FFR, and to assess whether lesion-specific FAI could enhance the diagnostic ability for functional ischemia in combination with other coronary CTA parameters to facilitate clinical decision making.

## Method

### Study Design

This retrospective cross-sectional study included data of patients scanned between April 2017 and October 2019 at seven centers (Shengjing Hospital of China Medical University, Shenyang, China; Guangdong General Hospital, Guangzhou, China; Shanghai Jiao Tong University Affiliated Sixth People's Hospital, Shanghai, China; Beijing Anzhen Hospital, Beijing, China; Peking Union Medical College Hospital, Beijing, China; The First Affiliated Hospital of China Medical University, Shenyang, China; The People's Hospital of Liaoning Province, Shenyang, China). The inclusion criteria were as follows: (1) patients with suspicion of CAD and underwent coronary CTA as part of the clinical routine; (2) an invasive coronary angiography (ICA) in combination with FFR measurement was performed within 1 week in a non-emergent setting. The exclusion criteria were as follows: (1) poor image quality (such as significant artifacts in the interested segment) or too limited or minimal PCAT to be analyzed (the thickness of PCAT was less than the diameter of the adjacent artery); (2) patients with anatomic variations in the heart or coronary arteries (significant abnormalities in the location of coronary ostium or severe myocardial bridging that might affect the lesion-related FFR measurement); (3) patients with previous CAD history, such as previous percutaneous coronary intervention, coronary artery bypass graft surgery or myocardial infarction; (4) patients with total occlusion lesion/s; (5) patients with malignancy or cardiomyopathies.

This retrospective study was approved by the institutional review board of Shengjing Hospital of China Medical University (No. 2021PS689K). Because it was a retrospective study and the examination was necessary for the clinical diagnosis of the subjects, the exemption of written informed consent was approved by the institutional review board.

**Figure 1:**
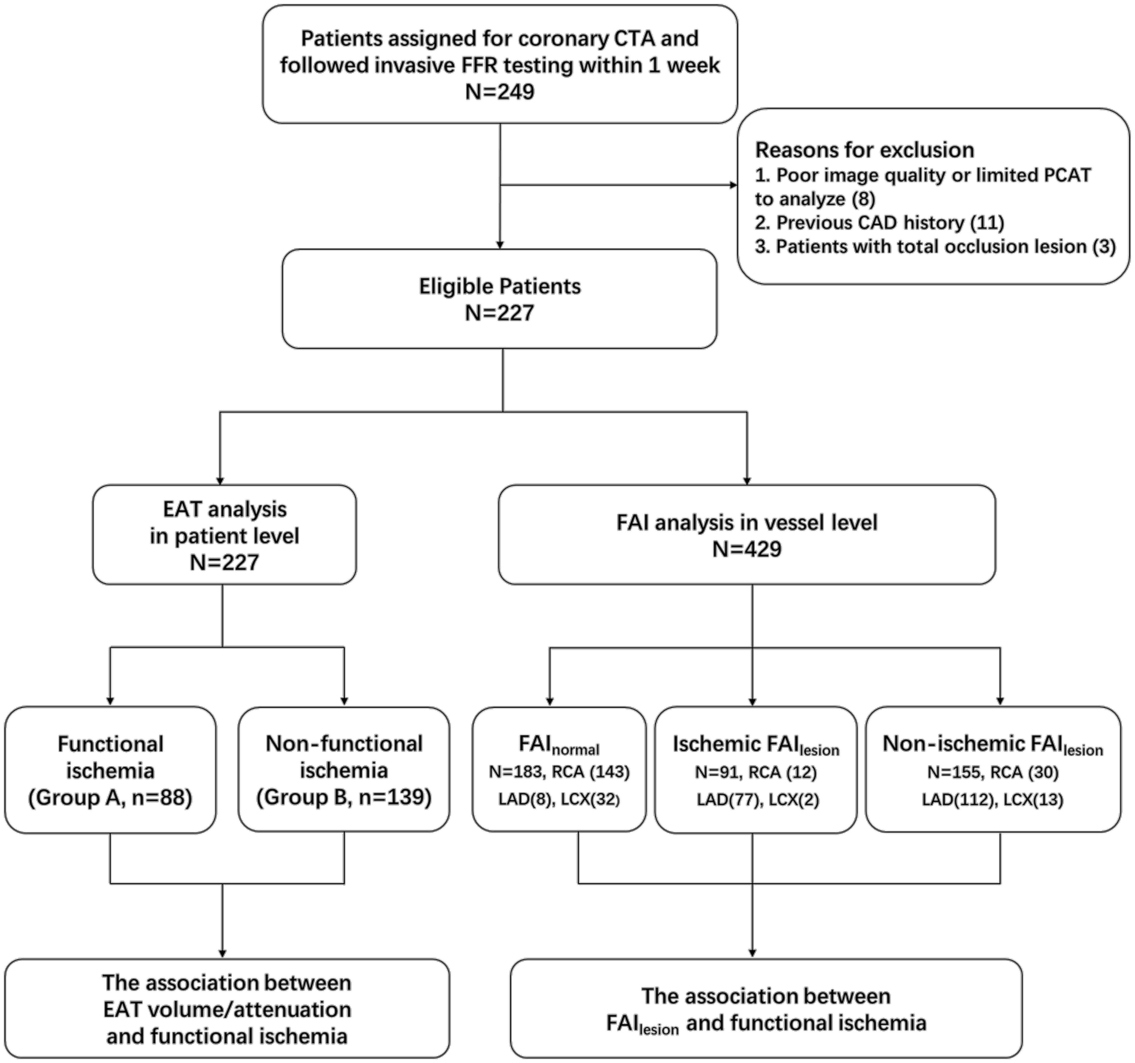
Study design and subjects grouping. CTA, computed tomography angiography; FFR, fractional flow reserve; EAT, epicardial adipose tissue; PCAT, peri-coronary adipose tissue; FAI, fat attenuation index; FAI_normal_, lesion-free fat attenuation index; FAI_lesion_, lesion-specified fat attenuation index.

All coronary CTA data were reviewed by a single experienced observer (with over 10 years experience) who was blinded to clinical data of the patients. All patients were divided into functional ischemia group (Group A) and non-functional ischemia group (Group B) based on the invasive FFR measurements. The study design and grouping scheme were shown in Figure 1.

### CT Acquisition and Reconstruction

All CTA scans carried out by each center were in accordance with the guidelines recommended by the Society of Cardiovascular Computed Tomography (25). All of the scans were performed with ≥64 slice multidetector scanners (Brilliance iCT, Philips Healthcare, Cleveland, OH, USA; Somatom Definition AS, Siemens, Forchheim, Germany; Somatom Definition Flash, Siemens Healthcare, Forchheim, Germany) with retrospective or prospective ECG gating. The general scanning parameters were as follows: collimator width of (2 × 64)/128/ 320 × 0.5/0.625 mm; tube potential of 120 kV; effective tube current of 400 to 700 mA (26). Contrast media (Omnipaque, 350 mgI/ml, GE Healthcare; Visipaque 320 mg/dl, GE Healthcare; Iopromide, 370 mgI/ml, Bayer) was injected with a flow rate of 4.5 ml/s (<80 kg body weight) or 5 ml/s (≥80 kg body weight) followed by a 30 ml saline flush. The total amount of contrast media was calculated by patient weight × 0.8 ml/kg. Before the CTA scanning, patients with HR>70 bpm were administered oral ß-receptor blockers (25 mg metoprolol succinate sustained-release tablets, AstraZeneca, Sweden).

### FAI Quantification

Peri-coronary adipose tissue is a part of EAT and is defined as the adipose tissue voxels [−190 to −30 Hounsfield units (HU)] located within a distance from the outer vessel wall equal to the diameter of the respective vessel (19). A semi-automated offline workstation (Dr. Wise® Coronary Artery CT Aided Diagnosis Software V200831, Deepwise Healthcare, Beijing, China) was used to measure the FAI. First, the plaques and stenosis were detected with deep learning algorithms based on multi-view CPR images (manual correction was performed if necessary). Then, PCAT was sampled radially outwards from the outer vessel wall, which was segmented using a multi-scale convolution neural network (CNN) with global vessel structure awareness. Finally, the sampled PCAT voxels were mapped into color-coded images and the FAI was calculated subsequently.

To conduct the analysis of FAI according to the lesion status of a vessel, we measured the lesion-specific FAI (FAI_lesion_) and lesion-free FAI (FAI_normal_), respectively. Lesion-free PCAT was defined as adipose tissue surrounding coronary arteries without atherosclerosis in the proximal segment of the Right Coronary Artery. If the RCA was atherosclerotic, PCAT of the left anterior descending (LAD) artery or the left circumflex (LCX) artery was used to measure FAI_normal_.

To assess the repeatability of FAI_lesion_ measurement, 30 patients were randomly selected with SPSS random number generator and assigned to another radiologist (over 5 years of experience) for FAI_lesion_ measurement.

### Quantification of EAT Volume and Attenuation

Epicardial adipose tissue volume and attenuation measurement was performed on an offline workstation (Cardiac Risk Assessment, version 1.2.0, Siemens Healthineer, Germany). EAT was defined as adipose tissue contained within the visceral pericardium. The visceral pericardium was identified and traced manually on axial CT images between the levels of pulmonary trunk bifurcation and cardiac apex. EAT volume was automatically calculated (in ml) using contiguous voxels with threshold attenuation of −190 to −30 HU (22) as the range for defining adipose tissue. Mean attenuation (HU) in the entire region of interest was obtained simultaneously and defined as EAT attenuation.

### Coronary Plaque Analysis

The plaque characterization was performed and a series of quantified plaque features were measured with a dedicated plaque analysis software (Coronary Plaque Analysis, version 1.2.0, Siemens Healthineer, Germany). Maximum diameter stenosis (DS), total plaque volume (TPV), and remodeling index (RI) were recorded using a method described previously (24). Plaques were further divided into their different components: low- (−30 to 30 HU), intermediate- (31–130 HU), and high-attenuation plaque (>130 HU), respectively. Each component was recognized and quantified automatically by the software and manual correction was performed if necessary. The volumes of calcified and non-calcified plaque were quantified as previously described (27). A high-risk plaque was identified when at least 2 of the following features were met: positive remodeling (≥1.1); low-density plaque (<30 HU); spotty calcification (<3.0 mm); or a “napkin-ring” sign (6, 27).

### Invasive Coronary Angiography and Fractional Flow Reserve

Selectively invasive coronary angiography (ICA), stenosis assessment, and FFR measurement were performed in each center by an experienced interventional cardiologist who was blinded to the CTA results. Standard quantitative coronary analysis (QCA) was used to quantify the ICA stenosis severity with the method as previously described. FFR was performed with a coronary pressure wire (Volcano, Rancho Cordova, CA, USA or St Jude Medical, Minneapolis, MN, USA) subsequently in vessels with intermediate stenosis between 30 and 90% (14, 16, 26). Maximum hyperemia was induced by intravenous administration of adenosine triphosphate (140–180 μg/kg/min). A stenosis>90%, or an FFR <0.80, was considered functional ischemia (15, 16, 24).

### Statistical Analysis

Data were analyzed using commercially available software (SPSS version 20.0, IBM Corp., Armonk, NY, USA and MedCalc Statistical Software, version 19.0.4, MedCalc Software Ltd, Ostend, Belgium). The continuous variables were described by mean ± standard deviation or median (interquartile range). The categorical variable was expressed as a percentage.

Independent-sample *t*-test, Mann-Whitney *U*-test, and chi-square test were applied to compare the difference of EAT parameters, FAI_lesion_, FAI_normal_, plaque characteristics, and traditional CAD risk factors between patients (or vessels) with and without FFR abnormity. Paired *t*-test was performed to compare the intra-patient difference of FAI_normal_ and FAI_lesion_.

Univariate and multivariate logistic regression analyses were applied to evaluate the association between FAI_lesion_, plaque characteristics, ICA stenosis severity, and functional ischemia. The receiver-operating characteristic (ROC) curve was used to analyze the discriminatory power of each parameter for predicting coronary functional ischemia. To assess the repeatability of FAI_lesion_ measurement, inter-observer variability was evaluated using the intraclass correlation coefficient (ICC). A *p* < 0.05 was considered statistically significant.

## Results

### Patient Characteristics and the Comparison of EAT

There were 249 patients that underwent coronary CTA scanning, subsequent invasive angiography, and FFR testing. Twenty-two of them were excluded for various reasons (details of which are shown in Figure 1). Finally, a total of 227 patients were included. Patient characteristics are shown in Table 1. There were 238 vessels of these patients that had intermediate lesions (a 30–90% lumen stenosis identified by ICA) and the FFR measurement was applied in these vessels. In addition, eight vessels presented stenosis of over 90% by ICA. Altogether, the FAI of 246 lesions was measured.

**Table 1:** The patient characteristics and general epicardial adipose tissue (EAT) parameters in different groups.

	**Total**	**Group A**	**Group B**	***P*-value**
**Patients (** ***n*** **)**	227	88	139	
Age	62 ± 10	60 ± 10	62 ± 9	0.1
Male (%)	153 (67)	64 (73)	89 (64)	0.22
BMI, (kg/m^2^)	24 ± 5	23 ± 6	26 ± 4	0.11
**CAD risk factors (%)**				
Diabetes mellitus	61	24	37	0.9
Hypertension	135	53	82	0.89
Hypercholesterolemia	66	24	42	0.72
Smoking history	71	33	38	0.18
Family history	29	13	16	0.67
EAT volume (ml)	170 ± 66	180 ± 73	163.5 ± 60	0.07
EAT attenuation (HU)	−83 ± 10	−84 ± 6	−83 ± 7	0.54

*BMI, body mass index; CAD, coronary artery disease; EAT, epicardial adipose tissue; HU, Hounsfield unit*.

Patients were classified according to their ICA and FFR testing results. Briefly, 88 (39%) patients were identified as having coronary functional ischemia. Three of these patients had ischemic lesions in two separated coronary main branches (LAD, LCX, or RCA). Thus, a total of 91 vessels were identified as flow limiting. To evaluate the association between EAT volume/attenuation and functional ischemia, we analyzed these parameters in patients with (group A) or without (group B) functional ischemia. The results indicated that there were no significant differences in the EAT volume (180 ± 73 ml vs. 163 ± 60 ml, *p* = 0.07) and attenuation (−84 ± 6 HU vs. −83 ± 7 HU, *p* = 0.54) between the two groups of patients (Table 1). There was no difference in traditional CAD risk factors between patients in groups A and B either.

### The Analysis of Lesion-Free and Lesion-Specific PCAT Attenuation

**Figure 2:**
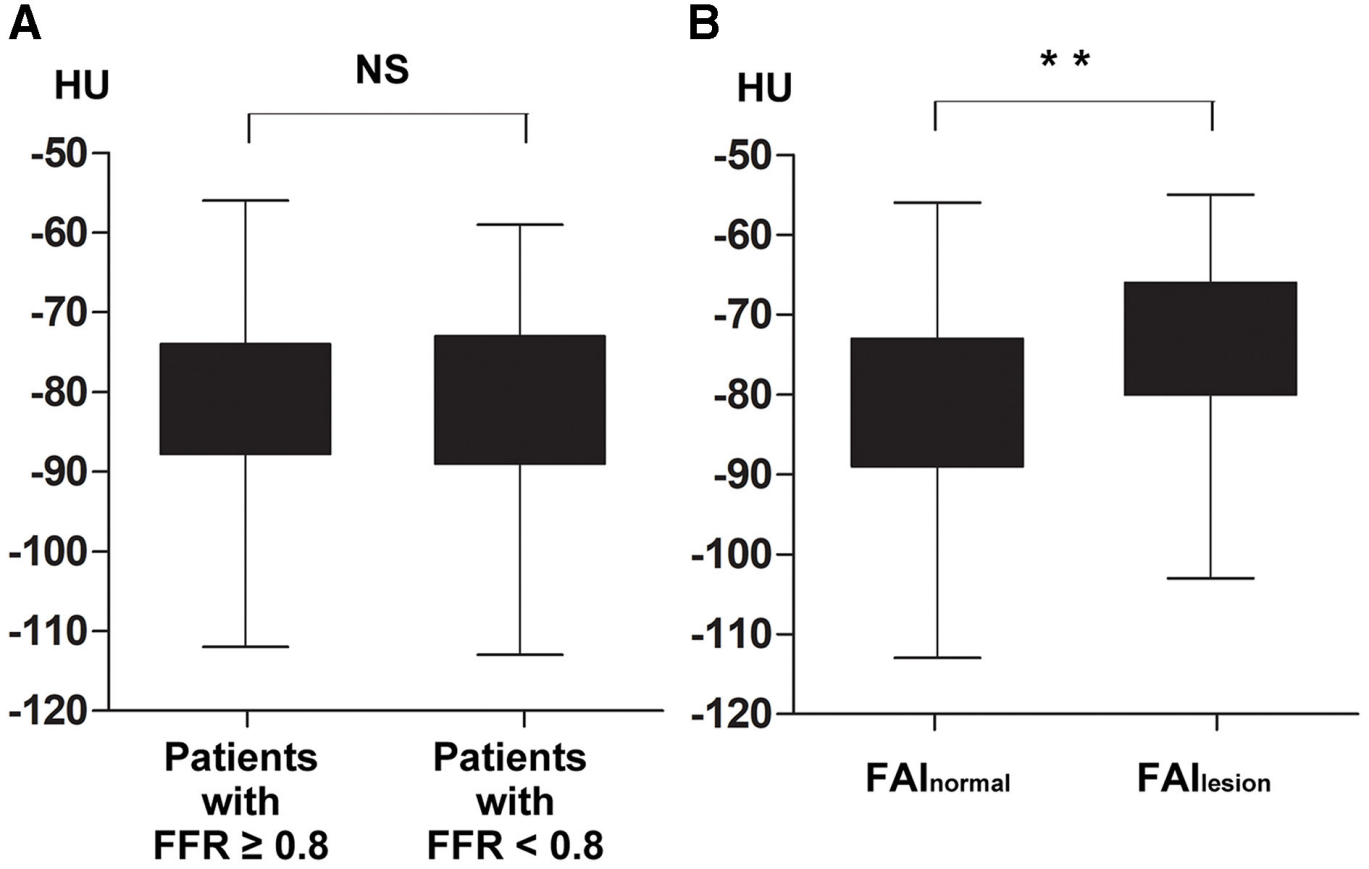
Comparisons of lesion-free fat attenuation index (FAI_normal_) in different kinds of patients and intra-patient comparison of FAI according to atherosclerosis status. There was no difference in FAI_normal_ between patients with and without functional ischemia, −82 ± 11 HU vs. −81 ± 11 HU, *p* = 0.65 **(A)**; FAI_lesion_ was significantly higher than FAI_normal_ with intra-patient analysis, 74 ± 9 HU vs. −81 ± 10 HU, *p* < 0.01 **(B)**. FAI_normal_, lesion-free fat attenuation index; FAI_lesion_, lesion-specific fat attenuation index; FFR, fractional flow reserve; HU, Hounsfield unit; NS, non-statistical significance. **p < 0.01.

To evaluate whether the PCAT of normal segments was different between patients with and without functional ischemia, the comparison of FAI_normal_ among patients with different ischemia statuses was performed. Atherosclerotic lesions were observed in proximal segments of all three coronary branches in 44 patients. Finally, 183 FAI_normal_ that were collected from 183 patients were included. Seventy-nine of the included FAI_normal_ came from patients with functional ischemia and the remaining 104 were from patients without functional ischemia. The result showed that there was no difference in FAI_normal_ between patients with and without functional ischemia (−82 ± 11 HU vs. −81 ± 11 HU, *p* = 0.65, Figure 2A).

**Figure 3:**
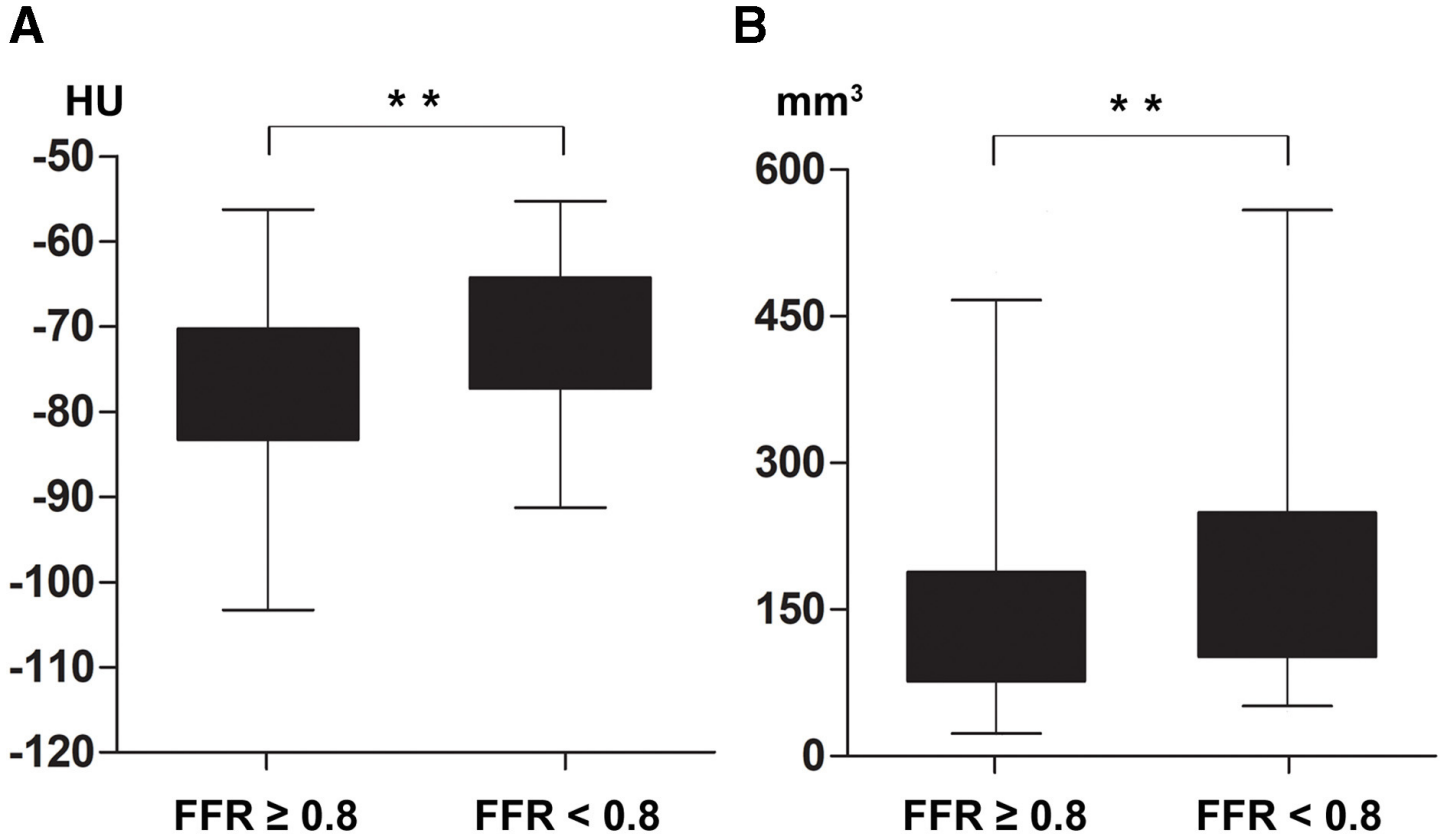
Comparisons of lesion-specified fat attenuation index (FAI_lesion_) and total plaque volume (TPV) in vessels with or without functional ischemia. Lesions with functional ischemia had higher FAI_lesion_, −71 ± 8 HU vs. −76 ± 9 HU, *p* < 0.01 **(A)**; and greater TPV, 157 (102, 248) mm^3^ vs. 126 (77, 188) mm^3^, *p* < 0.01 **(B)**. FAI_lesion_, lesion-specific fat attenuation index; TPV, total plaque volume; FFR, fractional flow reserve; HU, Hounsfield unit. **p < 0.01.

**Figure 4:**
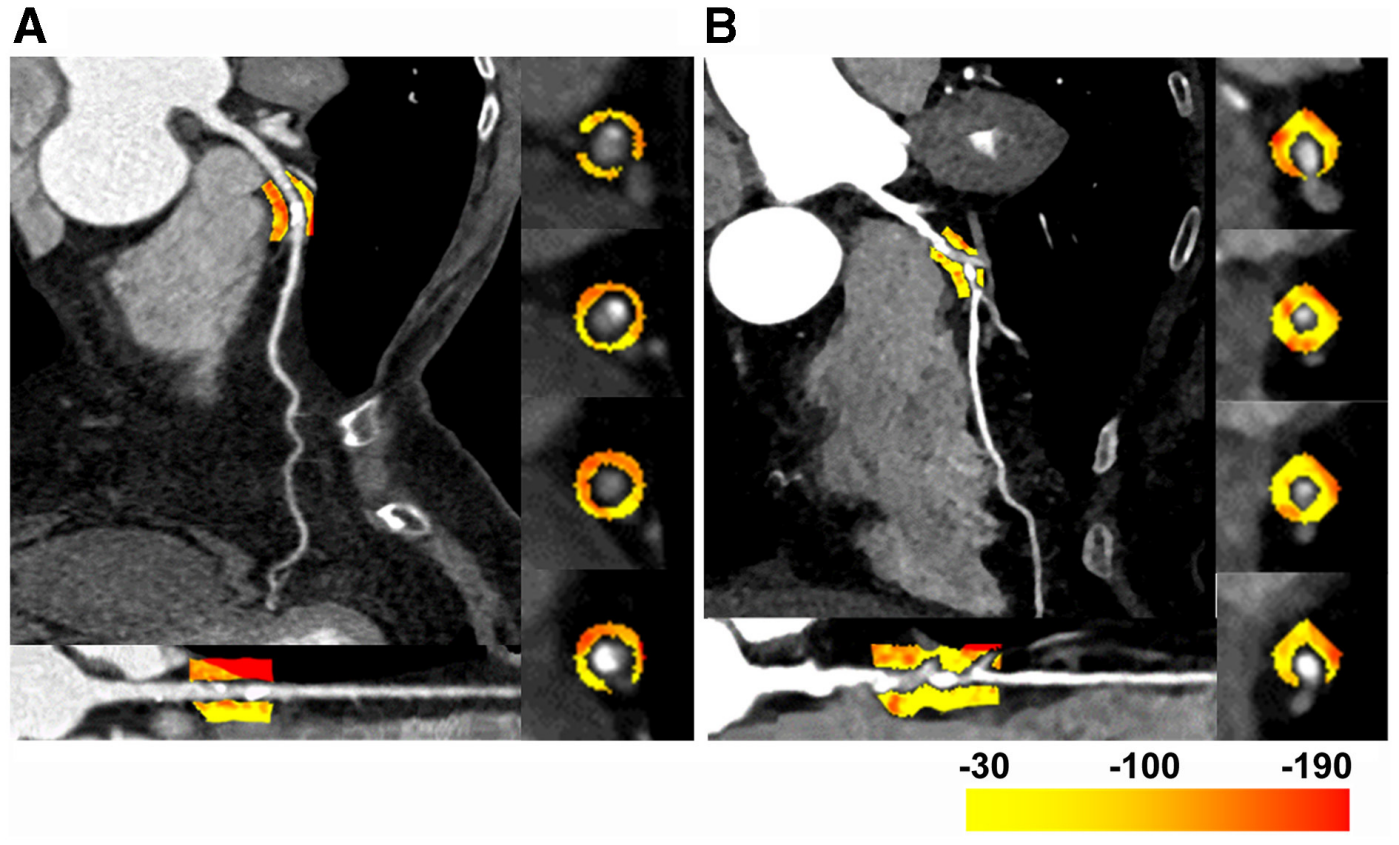
Two representative cases of the FAI difference in lesions with or without abnormal fractional flow reserve (FFR). **(A)** The left anterior descending (LAD) artery of a 60–65 years old patient. The FAI_lesion_ was −92 HU, and the FFR was 0.88 with a 70% lumen stenosis. **(B)** The LAD of a 70–75 years old patient. The FAI_lesion_ was −77 HU, and the FFR was 0.6 with a 78% lumen stenosis. FAI_lesion_, lesion-specific fat attenuation index; LAD, left anterior descending; FFR, fractional flow reserve; HU, Hounsfield unit.

To investigate whether FAI was different according to atherosclerosis and FFR status of the vessel, FAI of lesions that were performed FFR measurement or presented lumen stenosis over 90% were measured and analyzed. With intra-patient analysis including 183 paired FAI (FAI_lesion_ vs. FAI_normal_), the result showed that FAI_lesion_ was much higher than FAI_normal_ (−74 ± 9 HU vs. −81 ± 10 HU, *p* < 0.01, Figure 2B). And among the 246 lesion-specific PCAT, the FAI_lesion_ of ischemic lesions was significantly higher than that of non-ischemic lesions (−71 ± 8 HU vs. −76 ± 9 HU, *p* < 0.01, Figure 3A). Two representative cases were shown in Figure 4.

### Relationship Between Lesion Features, Lesion-Specific PCAT, and Functional Ischemia

The plaque causing functional ischemia had a larger total plaque volume [157 (102, 248) mm^3^ vs. 126 (77, 188) mm^3^, *p* < 0.01, Figure 3B], and more severe stenosis in both ICA (72 ± 9% vs. 56 ± 12%, *p* < 0.01) and CTA (66 ± 13% vs. 44 ± 22%, *p* < 0.01) testing. The differences of each plaque component ratios and high-risk plaque features between lesions with or without functional ischemia were non-significant (Table 2). With univariate logistic regression, the severity of stenosis (both ICA and CTA), TPV, non-calcified plaque volume, and FAI_lesion_ were shown to be directly correlated with abnormal FFR. In the multivariate analysis that included coronary CTA parameters (severity of stenosis, TPV, non-calcified plaque volume, high-risk plaque, and FAI_lesion_), we found that severity of stenosis and FAI_lesion_ were independently associated with abnormal FFR (Table 3).

**Table 2:** The comparison of plaque characteristics and lesion-specified fat attenuation index (FAI_lesion_) in different lesions.

	**Total**	**FFR <0.8**	**FFR ≥ 0.8**	***P*-value**
Vessels (*n*)	246	91	155	
**ICA stenosis, %**	62 ± 3	72 ± 9	56 ± 12	<0.01
<50% (*n*)	41	0	41	<0.01
50–70% (*n*)	132	39	93	
>70% (*n*)	73	52	21	
CTA stenosis, %	52 ± 22	66 ± 13	44 ± 22	<0.01
**TPV (mm** ^**3**^ **)**	134	157	126	<0.01
	(87, 208)	(102, 248)	(77, 188)	
HD-P volume (mm^3^)	60 (20, 113)	82 (31, 132)	48 (16, 100)	0.02
HD-P ratio, %	50 (17, 73)	50 (20, 74)	51 (12, 73)	0.82
ID-P volume (mm^3^)	52 (27, 99)	58 (33, 110)	50 (24, 87)	0.08
ID-P ratio, %	39 (23, 58)	37 (22, 54)	37 (25, 62)	0.31
LD-P volume (mm^3^)	9 (1, 19)	11 (1, 21)	7 (1, 16)	0.05
LD-P ratio, %	6 (1, 12)	6 (1, 12)	5 (1, 12)	0.47
Non-calcified plaque	64.4	69.4	59.4	0.04
volume (mm^3^)	(31.2, 118.9)	(39.0, 139.9)	(27.7, 115.1)	
**High-risk plaque features**			
Low-attenuation plaque	28 (11.4%)	13 (14.3%)	15 (9.7%)	0.27
Napkin-ring sign	20 (8.1%)	7 (7.7%)	13 (8.4%)	0.85
Spotty calcification	61 (25.2%)	19 (20.9%)	42 (27.1%)	0.26
Positive remodeling	139 (56.6%)	46 (50.5%)	93 (60.0%)	0.15
Lesion with high-risk plaque	53 (21.5%)	24 (26.4%)	29 (18.7%)	0.16
FAI_lesion_ (HU)	−74 ± 9	−71 ± 8	−76 ± 9	<0.01

*FAI_lesion_, lesion-specified fat attenuation index; ICA, invasive coronary angiography; CTA, computed tomography angiography; TPV, total plaque volume; HD-P, high-attenuation plaque component; ID-P, intermediate-attenuation plaque component; LD-P, low-attenuation plaque component; HU, Hounsfield unit*.

**Table 3:** Univariate and multivariate analysis for the prediction of functional ischemia.

	**Univariate analysis**			**Multivariate analysis**		
	**β coefficient**	**OR (95% CI)**	***P*-value**	**β coefficient**	**OR (95% CI)**	***P*-value**
TPV	0.004	1.004 (1.002–1.006)	<0.01	0.002	1.002 (0.998–1.006)	0.45
Non-calcified plaque volume	0.004	1.004 (1.001–1.008)	0.01	−0.002	0.998 (0.998–1.004)	0.58
High-risk plaque	0.44	1.56 (0.84–2.88)	0.16	0.24	1.27 (0.59–2.72)	0.54
FAI_lesion_	0.060	1.07 (1.04–1.11)	<0.01	0.060	1.08 (1.04–1.12)	<0.01
CCTA stenosis	0.072	1.06 (1.04–1.08)	<0.01	0.074	1.06 (1.04–1.08)	<0.01
ICA stenosis	0.13	1.13 (1.10–1.17)	<0.01	NA	NA	NA

*FAI_lesion_, lesion-specified fat attenuation index; TPV, total plaque volume; ICA, invasive coronary angiography; CCTA: coronary computer tomography angiography; OR, odds ratios; NS, no statistical significance; NA, not applied*.

### Diagnostic Performance of FAI and CTA/ICA-Derived Morphological Assessing for Abnormal FFR

**Figure 5:**
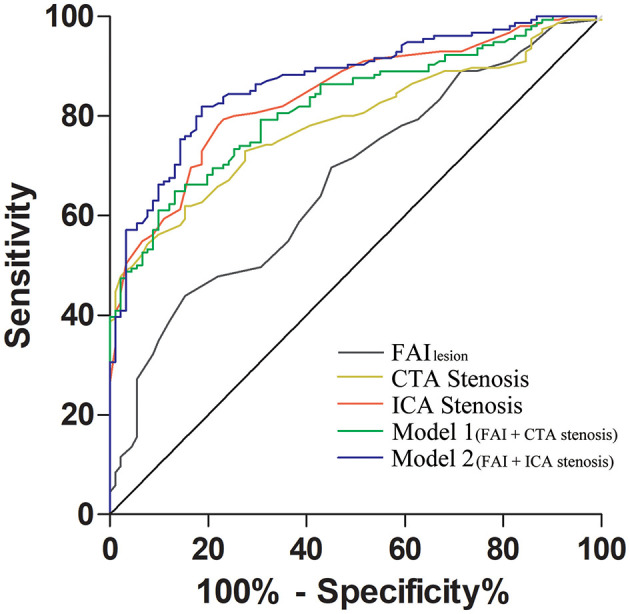
Comparison of the receiver operating characteristic (ROC) curves between different predictors. FAI_lesion_ + computed tomography angiography (CTA) stenosis had a similar AUC as invasive coronary angiography (ICA) stenosis alone (0.820 vs. 0.839, *p* = 0.39); FAI_lesion_ could also provide incremental diagnostic value to ICA stenosis (0.869 vs. 0.839, *p* = 0.03). AUC, area under the curve; FAI_lesion_, lesion-specified fat attenuation index; CTA, computed tomography angiography; ICA, invasive coronary angiography; Model 1, FAI_lesion_ + CTA stenosis; Model 2, FAI_lesion_ + ICA stenosis.

According to the ROC curve analysis, ICA stenosis severity had a higher AUC compared with either CTA stenosis severity or FAI_lesion_ for diagnosing functional ischemia. When integrating CTA stenosis and FAI_lesion_ (Model 1), the AUC of these combined non-invasive parameters increased and presented diagnostic performance similar to ICA stenosis (AUC: 0.820 vs. 0.839, *p* = 0.39). Combining FAI_lesion_ and ICA stenosis (Model 2), AUC was further improved beyond ICA stenosis alone (0.869 vs. 0.839, *p* = 0.03). Details of ROC analysis were shown in Table 4 and the comparison of AUC was shown in Figure 5.

**Table 4:** Diagnostic performance of coronary computed tomography angiography (CCTA)-derived parameters for predicting functional ischemic stenosis when using best cutoff values.

	**Cutoff value**	**AUC (95% CI)**	**Sensitivity, % (95% CI)**	**Specificity, % (95% CI)**	**Accuracy (%)**
CTA stenosis	>51%	0.784 (0.727–0.834)	84.62 (75.5–91.3)	61.69 (53.5–69.4)	68.98%
FAI_lesion_	>-79	0.674 (0.611–0.732)	84.62 (75.5–91.3)	44.16 (36.2–52.4)	64.90%
ICA stenosis	>66%	0.839 (0.787–0.883)	76.92 (66.9–85.1)	79.22 (72.0–85.3)	76.73%
Model 1	>0.3049	0.82 (0.766–0.866)	86.81 (78.1–93.0)	64.94 (56.8–72.4)	74.29%
Model 2	>0.4003	0.869 (0.821–0.909)	81.32 (71.8–88.7)	81.82 (74.8–87.6)	80.00%

*FAI_lesion_, lesion-specified fat attenuation index; ICA, invasive coronary angiography; CTA, computed tomography angiography; Model 1, FAI_lesion_ +CTA stenosis; Model 2, FAI_lesion_ +ICA stenosis*.

The ICC of inter-observer FAI_lesion_ measurement was 0.87 (95% CI:0.81–0.90) for average measures.

## Discussion

In the present study, we found that patients with or without functional ischemia caused by CAD (as determined by invasive FFR) might share similar EAT features and lesion-free PCAT attenuation. In contrast, lesion-specific FAI was independently associated with reduced FFR. Although the diagnostic performance of FAI_lesion_ or CTA lesion stenosis severity was individually inferior to invasive stenosis severity measurement, the predictive value of the combination model using these two CTA parameters was significantly improved and comparable to the ICA stenosis measurement.

To assess whether the difference of lesion-specific FAI between ischemic or non-ischemic lesions resulted from the difference of general EAT status, the comparison of EAT volume/attenuation between patients with and without functional ischemia was conducted. Some investigations have shown that EAT volume was associated with myocardial ischemia detected by nuclear imaging (28, 29), but the relationship between EAT volume and abnormal FFR remained unclear. Romijn et al. (15) indicated that EAT volume could independently predict FFR dropping while other works arrived at contrary results (13, 14, 16). The reason for this debate could be explained by the patient population included in the studies. In the study by Romijn et al. (15), invasive FFR was performed in only 32% of patients since 55% of patients had a stenosis <30% in all coronary arteries, indicating a low prevalence of severe stenosis and a high variety of CAD severity in the study subjects. However, subjects of the rest studies had a higher prevalence of significant CAD and a lower variety of CAD severity as reflected by a higher FFR utilization rate. As all patients in the present study were performed FFR measurements, it might be reasonable that we didn't find the connection of EAT volume to functional ischemia. As CT attenuation was thought to represent the inflammatory status of adipose tissue (19), we further investigated whether general EAT attenuation was associated with abnormal FFR. But the results showed that the general EAT attenuation in patients with or without functional ischemia was not significantly different either. Both general volume and attenuation could be used to present the global status of EAT. In patients with a relatively wide variety of lesion severity, the difference in their general statuses could be significant. Thus, the global information of EAT could be helpful for patient classification as shown in a previous study (15). Nevertheless, in a specific patient population with similar CAD severity (such as patients with intermediate stenosis who might share similar global EAT status), the general EAT parameters exhibited no significant difference (13, 14, 16). In such circumstances, the parameter that could represent focal coronary status might be helpful for patient/lesion classification.

Instead of EAT, the PCAT was taken to be a better indicator of the development of coronary atherosclerosis and the FAI had been proven to be associated with not only vessel inflammation but also patient's prognosis (19, 20). Studies had indicated that vessel-specific FAI measurement was associated with distal FFR dropping (9, 23), but the measured segment was not selected according to the atherosclerosis status of the vessel (the segments could be total-normal, total-lesion, or partial-lesion). The present study demonstrated that FAI_lesion_ was significantly higher than FAI_normal_. Thus, we assumed that vessel-specific FAI measurement could be affected by the ratio of normal to lesion segment in the measuring area, and lesion-specific FAI measurement might be a better indicator with lower variability. Two recent published studies used lesion-specific PCAT attenuation to predict abnormal FFR, but they reached contradictory results (16, 24). This could have been caused by their different measurement methods. Du et al. (16) manually measured peri-lesion EAT attenuation only within several circular regions of interest (ROIs) surrounding the study lesion on cross-sectional slices with the most severe diameter stenosis, and that would lead to high variability (and thus decreased reliability of the result). The present study supported the results that lesion-specific FAI was associated with abnormal FFR and could provide incremental diagnostic efficacy in combination with focal lesion characteristics (24). Our work had some advantages in comparison with the aforementioned studies. Besides a larger sample size collected from multiple centers, we set a baseline comparison of general EAT features and plaque-free FAI to prove the segment-to-segment interaction between PCAT and coronary lesions. Therefore, our study provided evidence of suitability and superiority of lesion-specific PCAT attenuation (FAI_lesion_) in comparison to general EAT parameters.

Studies have proposed that vascular inflammation was considered to be a contributor to endothelial dysfunction and would inhibit lipid accumulation in adipocytes of PCAT (19, 30, 31). Moreover, coronary atherosclerosis would not only increase immature adipocytes but may also lead to infiltration of pro-inflammation immunocytes within the adipose tissue (32, 33). With inflammation progressing, the fibrosis of adipose tissue would occur (34). All these changes of PCAT could affect the lipid/aqueous balance and lead to an increase in CT attenuation. Furthermore, these adverse changes of PCAT could cause an imbalance of anti or pro-inflammatory adipokines and cytokines that might have an influence on nearby vessels (35). Therefore, a vicious circle of inflammation might occur between atherosclerotic vessels and PCAT, and then aggravated endothelial dysfunction which caused impaired vasodilator capacity, which finally resulted in a relative pressure drop at maximal hyperemia induced by adenosine. Thus, this might explain the reason why FAI_lesion_ is higher for lesions with FFR <0.8.

Since the flow limiting caused by atherosclerosis mainly came with mechanical lumen stenosis, it was reasonable that CTA/ICA-assessed stenosis severity had higher diagnostic performance than FAI alone, which was treated as a focal inflammatory biomarker. However, the focal inflammatory could affect vasodilator function and further affect the distal pressure after coronary hyperemia. Therefore, it was rational that the diagnostic performance of morphological stenosis quantification (either assessed by CTA or ICA) could be improved by a focal inflammatory parameter (FAI_lesion_), as shown in the present results. Although stenosis severity assessed by coronary CTA was highly consistent with ICA, there were interference factors such as inappropriate intraluminal contrast enhancement, spatial resolution, and coronary calcification that would affect its accuracy (36). This could be the reason for the limitations of predicting abnormal FFR abnormity with CTA stenosis severity comparing with ICA.

As the lumen stenosis was the result of atheroma expansion and the expansion could either be concentric or eccentric, TPV could not independently predict ischemia. Whereas, the CREDENCE trial indicated that the percentage of non-calcified atheroma volume (plaque volume/vessel volume ×100%) and high-risk plaque features were independently associated with abnormal FFR (6). According to the CREDENCE trial, the percentage of atheroma volume (%) might be a better indicator for FFR in comparison with atheroma volume (mm^3^). Moreover, due to the inconsistent results of the individual plaque characteristic and high-risk plaque features on abnormal FFR (6, 24, 37, 38), further investigation might be in demand to explore the pathophysiological and hemodynamic effects of different plaque components on coronary flow limiting.

Our study indicated that the combination of FAI_lesion_ and CTA-assessed coronary stenosis severity had a much higher sensitivity than any other parameter for the diagnosis of functional ischemia lesions. That meant FAI_lesion_ and CTA-assessed stenosis severity, as a non-invasive method, could help us to rule out ischemic coronary stenosis more safely. As patient safety is the primary concern of clinical practice and the measurement of lesion-specified FAI does not require extra protocols or radiation exposure within routine coronary CTA, we believed that it was reasonable for quantifying lesion-specified FAI to facilitate clinical decision-making in patients with intermediate coronary stenosis assessed by CTA.

This study also had several limitations. First, as the enrolled subjects were clinically selected with intermediate lesions, they might not represent people with a low risk of CAD and severe diffuse coronary atherosclerosis. Second, based on the cross-sectional design, no causal relationship could be confirmed although multivariate regression analysis was performed. Moreover, no outcome data were included in the present study, and further research was needed to test the prognostic value of FAI_lesion_. Finally, because fixed tube voltage (120 kV) was applied in the present study and the attenuation of adipose tissue could be affected by the tube potential, the correction of HU value might be in demand for EAT/PCAT analysis if other scanning parameters were applied.

## Conclusion

Lesion-specific FAI measured by coronary CTA was independently associated with abnormal FFR and could improve the diagnostic performance in combination with morphological stenosis severity assessment. Therefore, the combined diagnostic model with FAI_lesion_ could be used to facilitate clinical decision-making and formulate therapeutic strategies.

## Data Availability Statement

The original contributions presented in the study are included in the article/supplementary material, further inquiries can be directed to the corresponding author/s.

## Ethics Statement

The studies involving human participants were reviewed and approved by the Institutional Review Board of Shengjing Hospital of China Medical University. Written informed consent for participation was not required for this study in accordance with the national legislation and the institutional requirements.

## Author Contributions

SM and YH contributed to conception and design of the study. HL, JZ, LX, YW, TL, and KW conducted the data collection. SM, XC, YM, and JY conducted the image analyzing and statistical analysis. SM wrote the first draft of the manuscript. All authors contributed to manuscript revision, read, and approved the submitted version.

## Funding

This study was granted by the National Natural Science Foundation of China (Grant Nos. of 82071920, 81901741, and 81871435); The Key Research & Development Plan of Liaoning Province (No. 2020JH2/10300037); 345 Talent Project in Shengjing Hospital of China Medical University.

## Conflict of Interest

The authors declare that the research was conducted in the absence of any commercial or financial relationships that could be construed as a potential conflict of interest.

## Publisher's Note

All claims expressed in this article are solely those of the authors and do not necessarily represent those of their affiliated organizations, or those of the publisher, the editors and the reviewers. Any product that may be evaluated in this article, or claim that may be made by its manufacturer, is not guaranteed or endorsed by the publisher.
